# Partitioned Stator Switched Flux Machine: Novel Winding Configurations

**DOI:** 10.3390/e22090920

**Published:** 2020-08-22

**Authors:** Muhammad Irfan, Naveed Ur Rehman, Faisal Khan, Fazal Muhammad, Abdullah S. Alwadie, Adam Glowacz

**Affiliations:** 1 Electrical Engineering Department, College of Engineering, Najran University Saudi Arabia, Najran 61441, Saudi Arabia; asalwadie@nu.edu.sa; 2 Department of Electrical Engineering, City University of Science and Information Technology, Peshawar 25000, Khyber Pakhtunkhwa, Pakistan; naveed.rehman@cusit.edu.pk; 3 Faculty of Electrical Engineering, Abbottabad Campus, COMSATS University Islamabad, Abbottabad 22060, Pakistan; faisalkhan@cuiatd.edu.pk; 4 Department of Automatic, Control and Robotics, AGH University of Science and Technology, 30-059 Krakow, Poland; adglow@agh.edu.pl

**Keywords:** HE-PSSFM3, finite element analysis, partitioned stator, permanent magnet, dual stator

## Abstract

Torque density is one of the major limiting factors in machine design. In this paper, we propose the hybrid excited partitioned stator switched flux machine3 (HE-PSSFM3). In HE-PSSFM3, armature winding is positioned on the outer stator whereas the permanent magnet (PM) and field winding are placed at the inner stator, while the rotor is free from excitation sources and armature winding. Moreover, concentrated field winding is replaced by toroidal winding. The power splitting ratio between two stators/rotor pole combinations is analytically optimized and are validated through genetic algorithm (GA) in order to enhance average torque and flux regulation capability. The electromagnetic characteristics of the improved and initial design are evaluated and compared with existing designs, i.e., HE-PSSFM1 and HE-PSSFM2. The proposed HE-PSSFM3 has achieved high average torque, i.e., 2.0015 Nm, at same armature and field current densities of 5 A/mm${}^{2}$. The results show that the average torques of the proposed design are 35% and 15% greater than HE-PSSFM1 and HE-PSSFM2, respectively. Furthermore, the analysis of various parameters such as flux linkage, flux regulation, electromagnetic performances, cogging torque, back EMF, electromagnetic torque, and torque ripples are investigated using two dimensional (2D) finite element analysis (FEA). Moreover, the simulation results of the proposed design are validated through GA and analytical modeling.

## 1. Introduction

Permanent magnet (PM) machines are distinguished by their improved efficiency and torque density [1,2]. Many researchers exploited the flux weakening capabilities of induction machine (IM) and their applications in variable speed electric vehicles, elevators, etc., [2]. In [3], wide constant speed power range of PM machine results from the flux weakening through injection of negative direct axis armature current is discussed. The applications of such type of interior permanent magnet (IPM) machines are very limited for variable speed systems due to their constant generated field. The hybrid excited (HE) machines use flux from both PM and field excitation coil. The high torque density, efficiency and wound field controllability of these machines are discussed in [4]. In [5], the ratio between PM flux and field winding flux as an additional degree of freedom is achieved. The output torque and high-power factor are maintained through the injection of a positive direct current in [6]. The HE machines improve the regulation of flux and inherits the benefits of PM machines [7]. The sliding contacts degrade the system thus effecting the reliability of system. In [8], the authors discuss drawbacks of machines having brushes and slip rings and thus, stator lodged field excited windings to address this issue. The fractional slot hybrid excited switched flux machine (HESFM) improves the flux weakening capabilities and are discussed in [9]. The armature current in PMs causes irreversible demagnetization. The direct axis and quadrature axis currents are separated to weaken the flux through armature currents. The capacity of armature current due to direct axis current is increased thus reducing the quadrature axis current and sacrificing the power factor hence torque capabilities [9].

HESFMs are more attractive in the research community due to; (a) high speed and greater torque density (b) constant power at wide range of speed and (c) fault tolerant capability [10,11,12]. In literature, various designs of HESFMs are classified as parallel and series flux paths [13,14,15,16,17]. PM machines have drawbacks of saturation and mechanical limitations. These problems are addressed by HEFSMs while used for high-speed applications, as there is no winding on rotor [18]. When a magnet is utilized in electric machine to pull ferromagnetic bits of metal out of a mixed system of materials, frictional and impact effects that necessarily occur increase surface temperatures leading to increases in thermal radiation. This increases energy dispersion, and energy dispersion is a sure indication of increasing entropy. Development of new rare-earth free permanent magnets has not been accomplished. Most of the high performance permanent magnet requires a combinational approach to alloy design and Dysprosium is one of the element to form neodymium-iron-boron (Neo) permanent magnets. Hence intense research is required to develop a highly-efficient permanent magnet based on the concept of highly entropy alloys (HEAs). As limited research is available in this area and HEAs permanent magnet are not utilized yet in the electric machine, reduction of rare-earth permanent magnet volume in electric machine is investigated from the last decade and performance of various topologies are examined. Space utilization is the major limitation of the HEFSMs. In [19], the authors investigate the hybrid excited partitioned stator switched flux machine-1 (HE-PSSFM1) where the PM volume is assumed 2500 mm${}^{3}$ at alternate stator pole, hence, it makes the machine cost effective [19]. However, due to less number of PMs the performance in terms of Power/torque density of the HE-PSSFM1 is still limited. To further improve the performance, the authors in [20] consider HE-PSSFM2 while increasing the volume of PM to 5000 mm${}^{3}$ as compared with HE-PSSFM1. With the increase in PM volume of HE-PSSFM2, the power/torque density increases, hence the cost also rises. In contrast to HE-PSSFM1 and HE-PSSFM2, a machine is required to keep the trade-off between the cost and torque density. In this paper, we propose a novel machine design named as HE-PSSFM3. In this paper short end winding and toroidal winding are used interchangeable, where the less number of PMs with toroidal winding is considered. In HE-PSSFM1 and HE-PSSFM2, the authors consider concentrated field winding with 6 and 12 PMs. In our proposed design i.e., HE-PSSFM3, with toroidal winding (short end winding) having 6 PMs is considered. The results indicate torque density of the proposed model is 35% and 15% higher than HE-PSSFM1 and HE-PSSFM2, respectively. Additionally, the analyses like flux linkage, flux regulation, electromagnetic performances, cogging-torque, back EMF, electromagnetic torque and torque ripples are investigated using 2D finite element analysis (FEA).

## 2. Design of Different Winding Configurations

**Figure 1:**
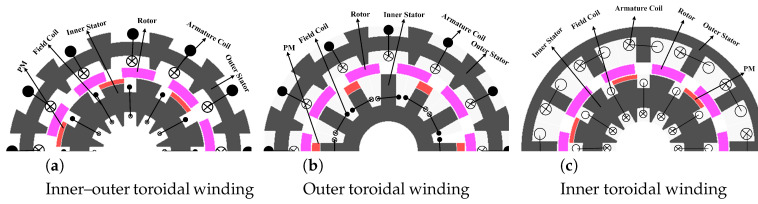
Structure view of different designs of winding configuration.

Figure 1 shows different winding configurations, (a) inner–outer, (b) outer and (c) inner toroidal winding arrangements. The toroidal winding has short end connection. The advantages of toroidal winding are to reduce copper losses, increase the efficiency, reduce volume and weight of the machine and is applicable for high-speed applications [21]. Performance analysis is carried out in this section in terms of electromagnetic torque, flux linkage and cogging torque of toroidal winding and is summarized in Table 1. The inner toroidal winding of flux linkage is 59% and 65% greater than outer and inner–outer toroidal windings, respectively. Similarly, the cogging torque of inner toroidal winding is 35% and 9% less than the outer and inner–outer toroidal winding. Moreover, the average torque of inner toroidal winding is 56% and 80% higher than outer and inner–outer toroidal winding, respectively.

## 3. Operation Principle and Machine Topology

**Figure 2:**
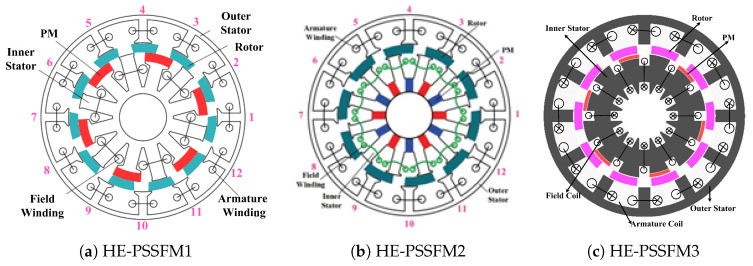
Proposed design HE-PSSFM3 and conventional designs i.e., HE-PSSFM1 and HE-PSSFM2.

Figure 2 illustrates the comparison of proposed HE-PSSFM3 with the HE-PSSFM1 [19] and HE-PSSFM2 [20]. Figure 2c depicts the HE-PSSFM3 with 10-Poles/12-Slots, that comprises of inner stator accommodating the toroidal field windings and PM, a piece of iron that is sandwiched between outer and inner stator and outer stator housing the armature windings, where the field excited coil (FEC) and armature windings are non-overlapping. The arrangement of armature coils is done in concentrated manner, while the FEC has toroidal winding configuration thus increasing the regulation of torque and flux density capability. The inner stator pole tip carries radially magnetized PMs and the identical slot number of outer and inner stator pole is used.

(1) $$\theta_{e}=N_{r}\theta_{m}$$

**Figure 3:**
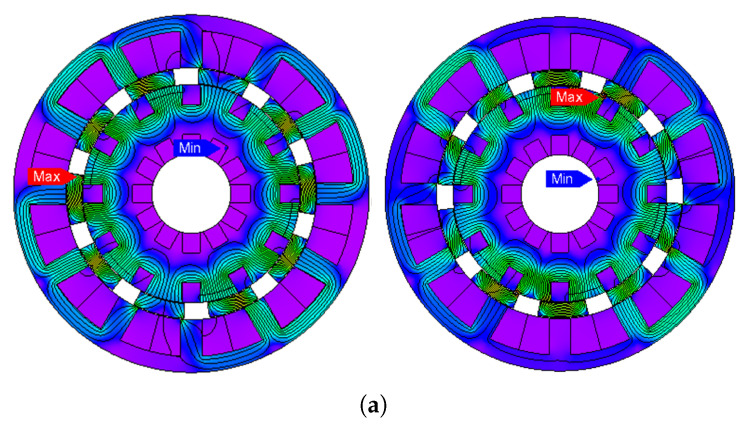

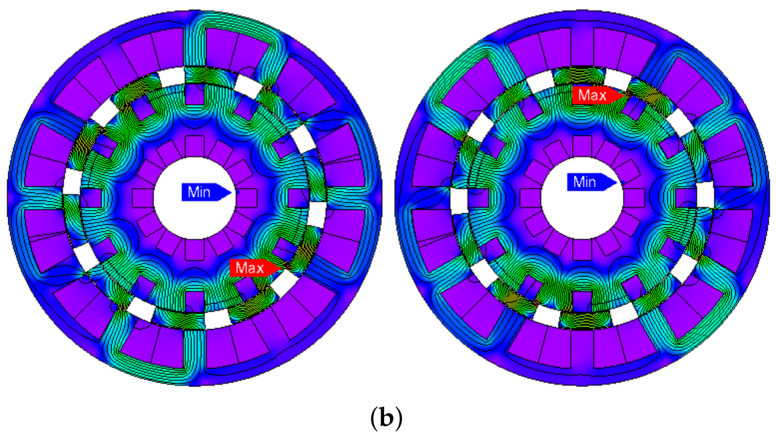
Flux distribution at different rotor positions by PM excitation only. (**a**) Electrical degree, $\theta_{e}=0^{\circ }$, $\theta_{e}=90^{\circ }$. (**b**) Electrical degree, $\theta_{e}=180^{\circ }$, $\theta_{e}=270^{\circ }$.

**Figure 4:**
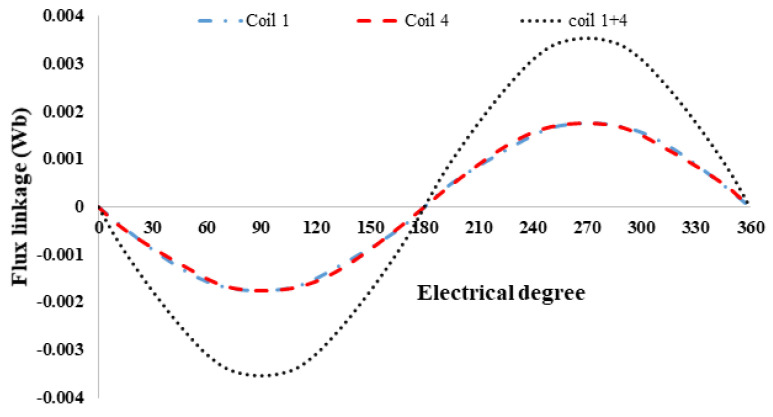
PM excitation only.

**Figure 5:**
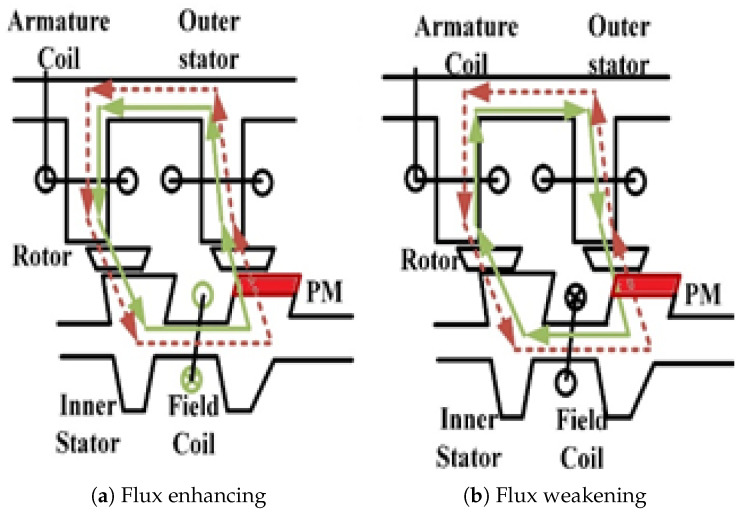
Paths of flux enhancing and flux weakening.

**Figure 6:**
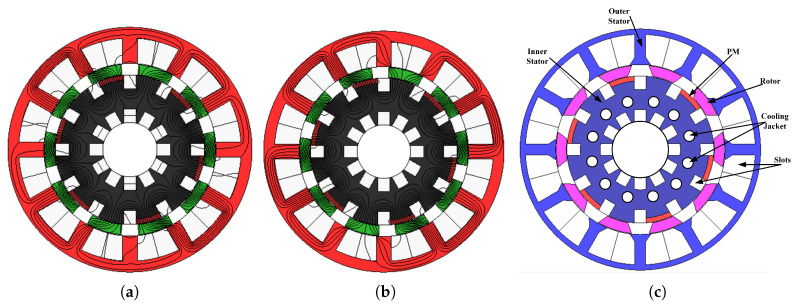
Flux distribution at no load/cooling jacket of inner stator HE-PSSFM3; (**a**) flux enhancing, (**b**) flux enhancing and (**c**) cooling jacket of inner stator HE-PSSFM3.

**Figure 7:**
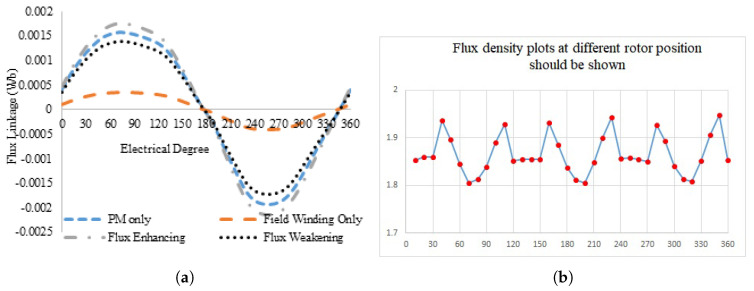
Flux linkage/flux density plots at different rotor position; (**a**) flux linkage with combined excitation, and (**b**) flux density plots at different rotor position.

The operating principles of HE-PSSFM2 and HEPSSFM3 are the same. The rotor electrical position $\theta_{e}$ is given as [20], where Nr, $\theta_{m}$ are the rotor pole number and rotor mechanical position. In the proposed HE-PSSFM3 design, in Figure 2c the coils 1, 4, 7 and 10 are combined in a forward direction. Figure 3 shows the open circuit flux distribution of HE-PSSFM3 at four different angle rotor positions. Figure 4 illustrates phase A flux linkage, which cut the peak point of flux at $0^{\circ }$ and $180^{\circ }$ and zero flux at $90^{\circ }$ and $270^{\circ }$. In addition, the combined coil 1 and 4 at no-load analysis is shown in Figure 4 and the result is near to pure sinusoidal. The result describes odd hormonic elimination in the machine. The design parameters are summarized in Table 2. Flux enhancing and flux weakening operations and regulating flux operation are specified principles of proposed design as shown in Figure 5. PMs and field excitation coil generated flux are added at the same time in the air-gap. In Figure 6, we observe the condition of flux enhancing and flux weakening at electrical degree, $\theta_{e}$ = $\theta_{o}$ rotor position. Cooling jacket concept is proposed for thermal management as shown in Figure 6c. The three types of techniques are radiation, convection and conduction. In this design, water is selected as the cooling medium. The temperature of water cannot increase because water enters from one side and out from the other side. A major part of heat is absorbed by the coolant medium and some of the heat will flow through convection to air [22]. Water jacket cooling system is available for a maximum current density of 30 A/mm${}^{2}$. Figure 7a shows the flux linkage at no-load analysis by applying 5 A/mm${}^{2}$ current density. Figure 7b shows the flux density plots at different rotor positions.

## 4. Electromagnetic Performance

**Figure 8:**
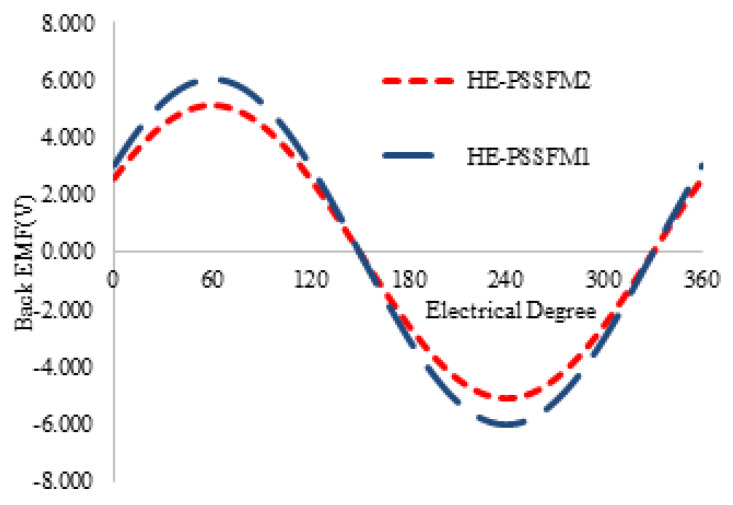
Back EMFs at 400 rpm without field excitation.

Figure 8 illustrates the induced-voltage of proposed design and existing designs HE-PSSFM2 and HE-PSSFM1 at a speed of 400 rpm at the no-load condition. The profile of back EMF maximum amplitude of is lower than existing design. The back EMF profile of HE-PSSFM3 is nearly sinusoidal, which has negligible odd harmonics as depicted in Figure 8.

### 4.1. Cogging Torque

**Figure 9:**
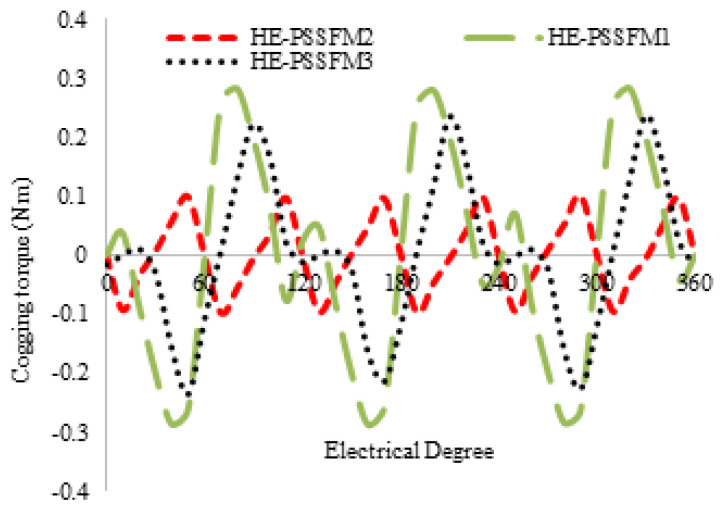
Cogging torque without field excitation.

Cogging torque is defined as unwanted phenomena in electrical machine design. The Figure 9 illustrate cogging torque of the proposed design is 52% and 25% greater than HE-PSSFM2 and HE-PSSFM1 respectively. By applying the genetic algorithm (GA) optimization technique, we minimize the peak points of cogging torque and enhance the average torque. Furthermore, to reduce by adjusting inner and outer pole arcs significantly reduced cogging torque, and has negligible effects on average torque.

### 4.2. Flux Regulation

**Figure 10:**
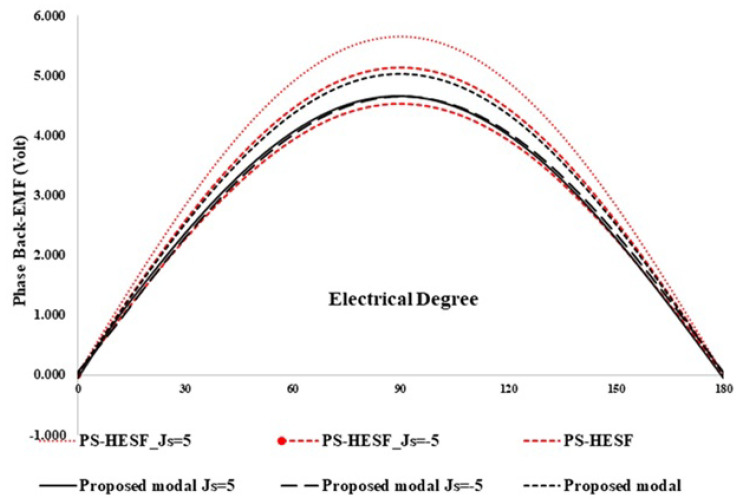
Back EMF waveform at 400 rpm with various field excitation.

**Figure 11:**
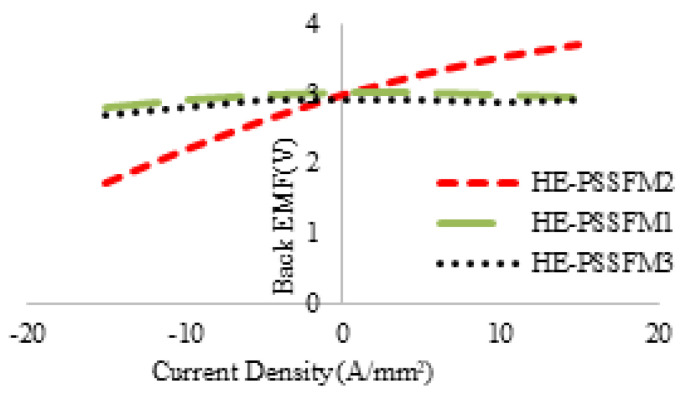
Variations of peak fundamental back EMF.

In this section, flux regulation is discussed which is a typical property of hybrid exited machines. Figure 10 shows the profile of back EMF with various field current density and constant speed at 400 rev/min. The armature current density is represented with Js = 5 and Js = −5 and Je of 5 A/mm${}^{2}$, with both positive and negative polarities. The back EMF profile of HE-PSSFM3 has larger variation range than existing the design because of the high area field coil slots. In Figure 11, the variation ranges of the proposed and existing designs are compared. Figure 11 shows that HE-PSSFM2 has higher variation range than HE-PSSFM1 and HE-PSSFM3 designs.

### 4.3. Torque

**Figure 12:**
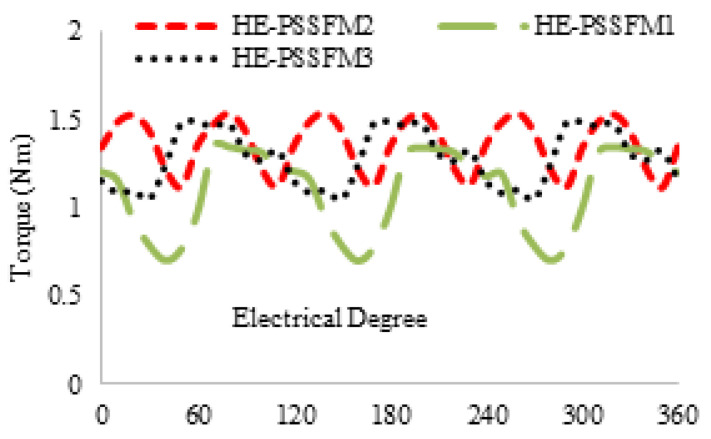
Torque waveforms with various field excitations (20 W copper losses).

Figure 12 shows the proposed design has an instantaneous torque at maximum current density 5 A/mm${}^{2}$. The proposed design has a higher average torque and less torque ripple than existing designs. However, generating high average torque is due to high slot area of armature and field windings and better space utilization of the machine. From Figure 12, it is observed that the maximum occurred at 0${}^{\circ }$ and that shows it had insignificant reluctance on torque. Table 3 summaries average torque at different field current densities.

The conventional designs are lower than the proposed design at PMs excitation and field enhancing conditions. The field current effect on average torque ($T_{avg}$) is critically observed. Figure 12 shows un-optimized torque of the proposed design and optimized torque of the existing design. The proposed design is less than HE-PSSFM2 and greater than HE-PSSFM1. The proposed torque is further improved by applying GA technique.

## 5. Optimization Procedure

### 5.1. Analytical Design Procedure of Power Splitting Ratio for Torque Maximization

Since there are various parameters in the HE-PSSFM3 machine, it is desirable to develop a simple analytical design procedure to facilitate the parametric optimization.

**Figure 13:**
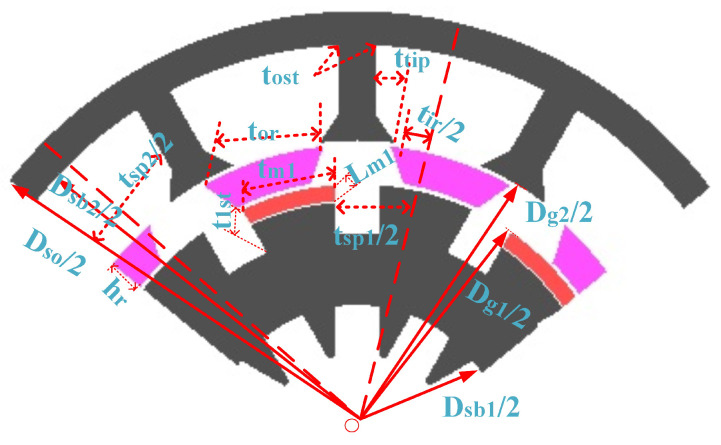
Design parameters in HE-PSSFM3.

The power splitting between two separate stators should be considered in advance. Figure 13 illustrates the geometric parameter of the proposed design. In reality, magnetic-thickness effects inner-slot width and therefore it is important for balancing the electric and magnetic loading because the excitation sources are housed in the inner-stator.

### 5.2. Optimal Electric Loading

The dominant sensitive parameter, effective split ratio ($\lambda_{s}$) is define as

(2) $$\lambda_{s}=\frac{D_{g1}}{D_{g2}}$$

Analytical method is implemented to aid the optimization of $\lambda_{s}$. As a result, according to [22], the average electromagnetic power delivered by the interaction between two sets of winding, is given by

(3) $$P_{o}=3E_{oa}I_{oa}\eta =3\pi^{2}B_{\delta_{m}}A_{o}D_{g2}^{2}L_{a},$$

(4) $$P_{i}=E_{ia}I_{ia}\eta =\pi^{2}B_{\delta_{m}}A_{i}D_{g1}^{2}L_{a},$$

where, $\frac{E_{ia}}{E_{oa}}$ and $\frac{I_{ia}}{I_{oa}}$ are the back EMF and phase current value of the inner and outer winding, $\frac{D_{g1}}{D_{g2}}$ are the diameter inner/outer air-gap, $L_{a}$ is the axial length, η is the efficiency, $B_{\delta_{m}}$ is the optimal flux density in air-gap and $\frac{A_{i}}{A_{o}}$ are the inner/outer electric loading. Hence, the total power can be expressed as

(5) $$P_{total}=P_{i}+P_{o}=\pi^{2}L_{a}\left(3A_{o}D_{g2}^{2}+A_{i}D_{g1}^{2}\right)$$

The electric loading of inner and outer air-gaps, Ai and Ao, respectively are shown as

(6) $$A_{i}=\frac{2K_{p}J_{si}A_{si}}{\pi D_{g1}}$$

(7) $$A_{o}=\frac{6K_{p}J_{so}A_{so}}{\pi D_{g2}}$$

Here, $K_{p}$, $J_{si}$, $J_{so}$, $N_{si}$ and $N_{so}$ are the slot-packing factor, the inner current density, the outer current density, number of outer stator slots and number of outer stator slots, respectively. The turn per phase in outer and inner winding can be calculated by

(8) $$J_{si}A_{si}k_{p}=N_{ia}I_{ia},$$

(9) $$J_{so}A_{so}k_{p}=N_{oa}I_{oa}.$$

By substituting (3) and (4) into (6) and (7), it yields

(10) $$P_{o}=3\pi B_{\delta_{m}}KpJ_{so}A_{so}D_{g2}L_{a},$$

(11) $$P_{i}=2\pi B_{\delta_{m}}KpJ_{si}A_{si}D_{g1}L_{a}.$$

The total copper loss is constrained in this case during the optimization, and hence the following constraint can be obtained

(12) $$\begin{array}{c} \left(L_{ia}+L_{iend}\right)\frac{{\left(N_{ia}I_{ia}\right)}^{2}}{A_{si}}+\left(L_{oa}+L_{oend}\right)\frac{{\left(N_{oa}I_{oa}\right)}^{2}}{A_{so}}\\ =\mathrm{Constant} \end{array}$$

(13a) $$\begin{array}{cc} K_{1} & =L_{ia}+L_{iend}, \end{array}$$

(13b) $$\begin{array}{cc} K_{2} & =L_{oa}+L_{oend}, \end{array}$$

(13c) $$\begin{array}{cc} K_{3} & =A_{si}, \end{array}$$

(13d) $$\begin{array}{cc} K_{4} & =A_{so}, \end{array}$$

(13e) $$\begin{array}{cc} K_{5} & =D_{g1}, \end{array}$$

(13f) $$\begin{array}{cc} K_{6} & =D_{g2}. \end{array}$$

Therefore, the total power extremism problems are equalized as the maximization of the term $K_{5}N_{ia}I_{ia}+K_{6}N_{oa}I_{oa}$ according to the Cauchy inequality theorem [23].

(14) $$\begin{array}{c} \left(\frac{K_{1}}{K_{2}}{\left(N_{ia}I_{ia}\right)}^{2}+\frac{K_{2}}{K_{4}}{\left(N_{oa}I_{oa}\right)}^{2}\right)\left(\frac{K_{3}}{K_{1}}K_{5}^{2}+\frac{K_{4}}{K_{2}}K_{6}^{2}\right)\\ \geq {\left(K_{5}\left(N_{ia}I_{ia}\right)+K_{6}\left(N_{oa}I_{oa}\right)\right)}^{2}. \end{array}$$

Based on Equation (13), the maximum torque density can be obtained when the following relation is satisfied

(15) $$\frac{N_{ia}I_{ia}}{N_{oa}I_{oa}}=\frac{K_{2}K_{3}k_{5}}{K_{1}K_{4}K_{6}}$$

In this case, the power split ratio can be calculated as

(16) $$Kpow=\frac{Po}{P_{i}}=\frac{3A_{os}D_{g2}}{2A_{is}D_{g1}}=\frac{3K_{4}K_{6}}{2K_{3}K_{5}}$$

**Figure 14:**
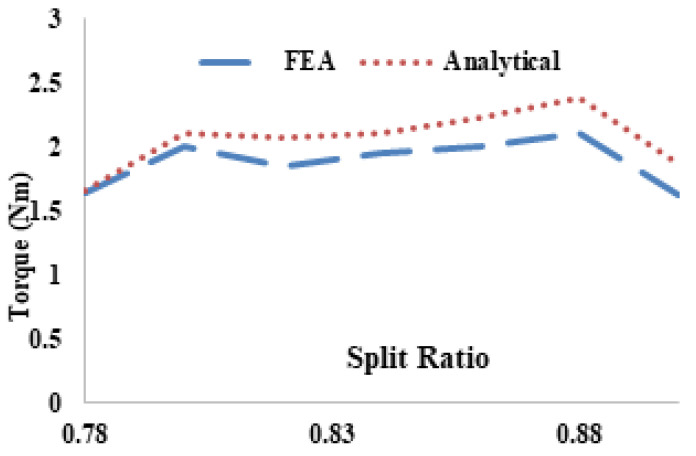
Variation of normalized torque with $\lambda_{s}$: Comparison between analytical and FEA predictions.

Figure 14 shows the comparison of FEA and analytical modeling of average torque with respect to split ratio. It is observed that FEA and analytical results are approximately the same and 0.86 is the maximum split ratio and error is less than 2.3%. The analytical technique fails to account end effect, non-linear magnetic saturation and flux linkage.

### 5.3. Genetic Algorithm

**Figure 15:**
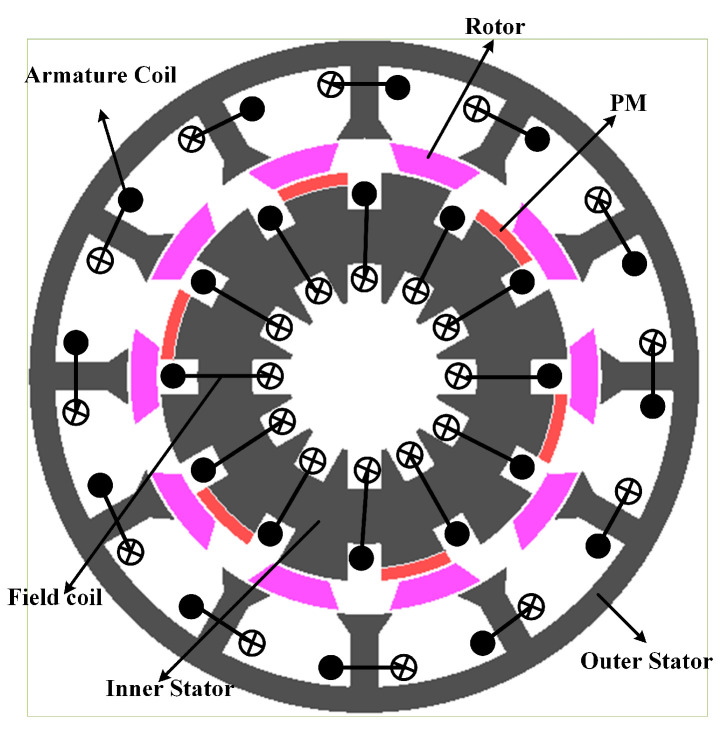
Geometric parameter/optimized design of HE-PSSFM3.

GA optimization technique is applied to HE-PSSFM3, which increases the performance in terms of flux linkage and reduced cogging torque. The HE-PSSFM3 has achieved better performance than existing HE-PSSFM1 and HE-PSSFM2. Figure 15 shows the geometric parameter/optimized design of the proposed machine. It should be emphasized that there is a trade off among the cogging torque, flux linkage and electromagnetic torque.

**Figure 16:**
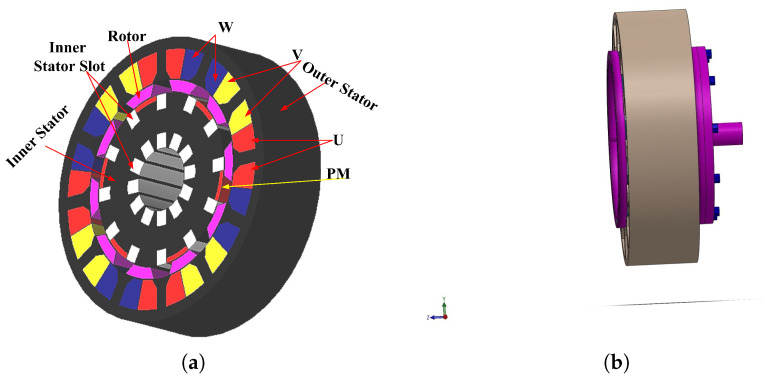
3D sketch/mechanical assembly of HE-PSSFM3; (**a**) 3D sketch, and (**b**) mechanical assembly.

**Figure 17:**
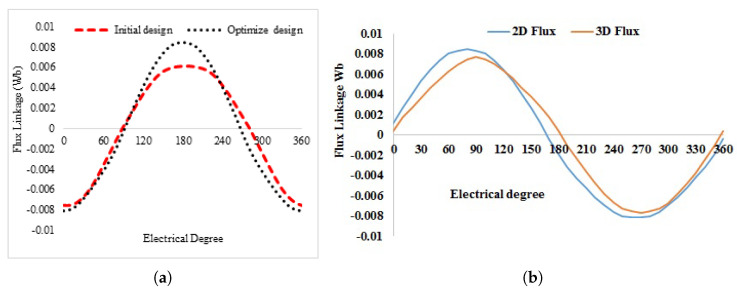
No-load flux linkage at PM excitation only; (**a**) flux linkage of initial and designs, and (**b**) flux linkage of 2D and 3D design.

**Figure 18:**
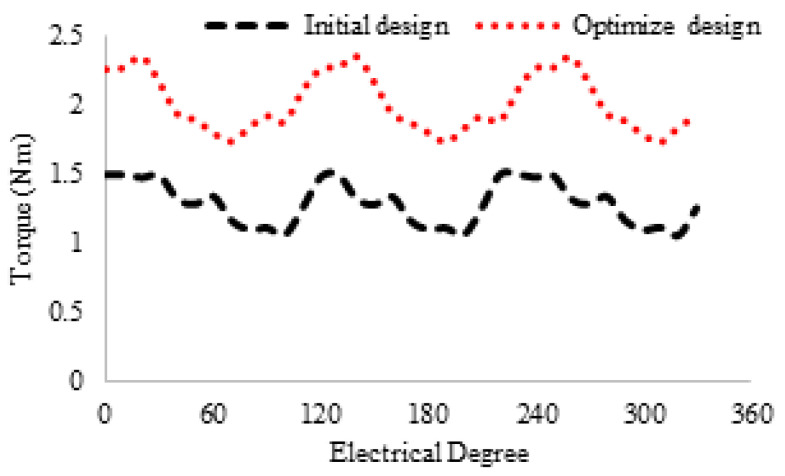
Torque waveforms with different field excitations.

**Figure 19:**
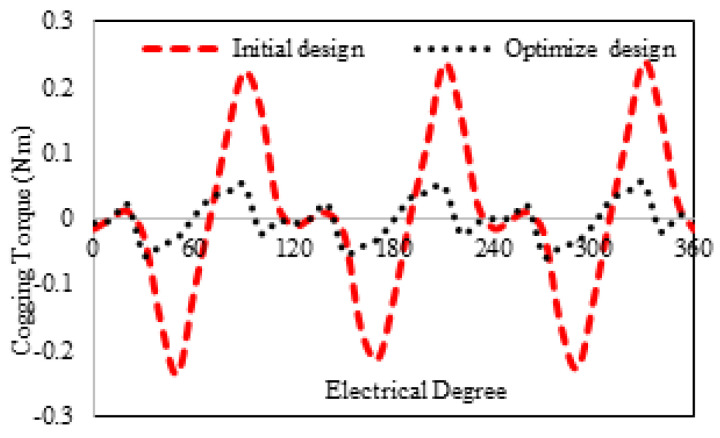
Cogging torque without field current of initial and optimized design.

Figure 16 shows the 3D sketch and mechanical sketch of the HE-PSSFM3. The characteristics of the proposed machine are enhanced by global optimization of rotor and PM. Table 4 summarizes the initial and final design parameters. After global optimization, the electromagnetic flux linkage is 33% greater than initial designs, as depicted in Figure 17a, similarly, the flux linkages of 2D and 3D are shown in Figure 17b. Moreover, the electromagnetic torque is 36% greater than the initial design and is depicted in Figure 18. Similarly, the peak-to-peak cogging torque reduced up-to 76.8% as is illustrated in Figure 19. Copper consumption is the key factor to be considered while designing the machine. Higher copper consumption causes three main drawbacks in the design. Firstly, large copper losses, secondly, high cost of machine and thirdly, low efficiency of machine. The proposed HE-PSSFM3 with non-overlapped and toroidal windings arrangement has less copper consumption and high efficiency. HE-PSSFM3 copper losses can be calculated using Equation (16).

(17) $$P_{cu}=I_{e}^{2}R_{e}+I_{a}^{2}R_{a}$$

**Figure 20:**
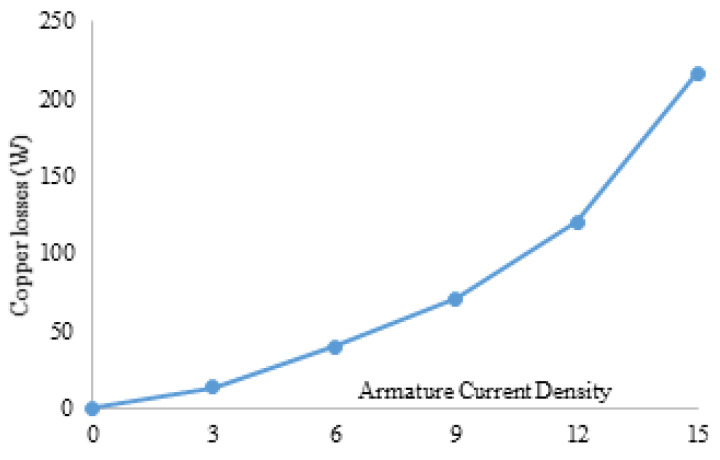
Copper losses at various current density.

**Figure 21:**
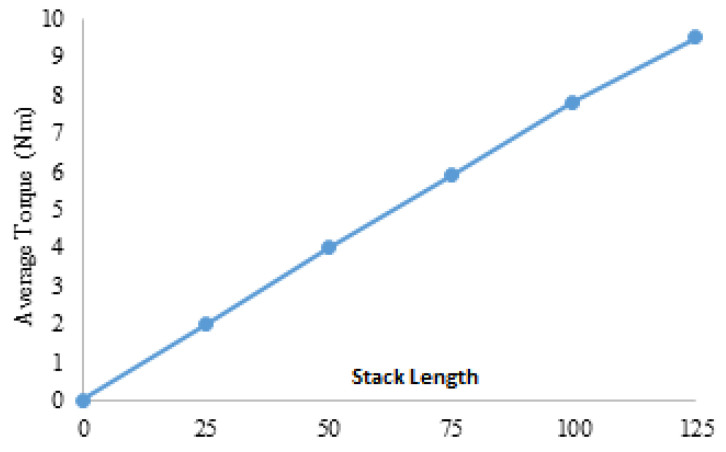
Stack length versus torque.

In Equation (16), R and I are the root mean square current and winding resistance, respectively, and subscripts “e” and “a” represent FEC and armature, respectively. Figure 20 illustrates copper losses verses armature current density of HE-PSSFM3 and maximum copper loss is 216.6 Watt at 15 A/mm${}^{2}$. Copper loss remains constant before and after optimization process because slot area un-changed during optimization. Figure 21 shows the stack length verses average torque at maximum armature and field current density and with constant speed. The average torque is approximately linearly increasing with increasing stack length.

## 6. Torque Comparison with HE-PSSFM1 and HE-PSSFM2 Machine

**Figure 22:**
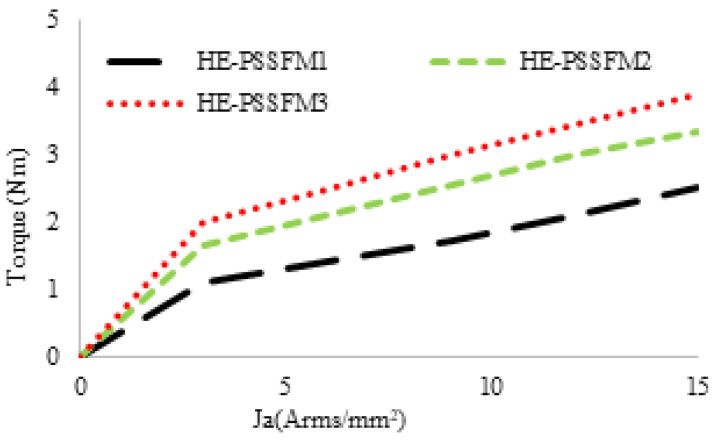
Torque versus armature current densities.

Figure 22, the average torque of proposed design, in compression with the HE-PSSFM1 and HE-PSSFM2 varies by changing the armature current density ($J_{a}$) while keeping field current density ($J_{e}$) constant. It is observed that electromagnetic torque is improved with an increase in $J_{a}$, and achieved an optimal torque of 3.89 Nm for HE-PSSFM3. The torque increases linearly which shows that there is no flux cancellation and saturation. The HE-PSSFM3 machine average electromagnetic torque is 35% and 13% greater than HE-PSSFM1 and HE-PSSFM2 machines, respectively.

## 7. Stress Analysis

**Figure 23:**
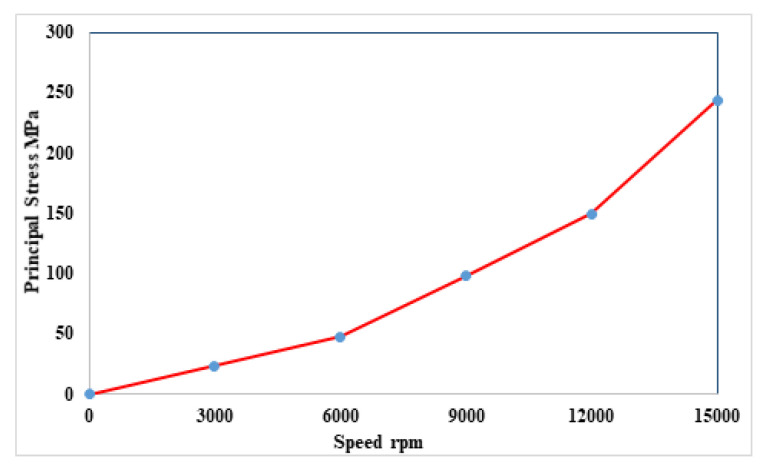
Stress verses speed.

The rotor stress analysis is very important for high-speed machines. It is identifying the optimal rotor tensile strength at different speeds. Beyond the maximum stress, the rotor structure breaks. Rotor stress analysis is a technique to identify the principal stress, nodal force and displacement occurring in the rotor structure in an ideal state after load is applied. Generally, the condition for mechanical stress of the rotor structure is accomplished by centrifugal force due to the longitudinal rotation of rotor. Additionally, centrifugal force of the rotor is greatly affected with the speed. The rotor could highly withstand the stress if the principal stress of the rotor is higher. Principal stress is a crucial result in the analysis of stress. With increasing the angular velocity of the rotor, principal stress is increased exponentially. 35H210 steel is used for outer rotor HE-PSSFM3, which has a maximum tensile strength of 500 MPa. Figure 23 shows stress analysis at various speeds. At 15,000 rpm, the rotor maximum stress is 243.5 MPa, which shows that it can rotate without any deformation at high speed.

## 8. Conclusions

In this work, we presented the design and analysis of HE-PSSFM3. This integration significantly reduced the space of the proposed design. We combine the partitioned stator design and dual stator design. The field winding and permanent magnet are housed in the inner stator where the armature winding is placed at the outer stator. More toroidal winding is used which has a short end connection, high torque density and is suitable for high-speed application. The proposed design has a better performance in terms of flux linkage and average electromagnetic torque than the conventional machines. The permanent magnet is reduced up to 50% and thus reduced the cost of the machine. The splitting ratio between two stators is analytically optimized and validated using GA. Consequently, it is assumed that FEA results represented in this paper are justified. Moreover, the thermal study and analytical modeling are interesting future extensions to the proposed design.

## Figures and Tables

**Table 1:** Comparisons of different winding configurations.

Characteristic	Unit	Different Winding Configuration		
		Inner Toroidal	Outer Toroidal	Inner and Outer Toroidal
Cogging torque	mNm	438.5	683.4	484.1
Flux linkage	mWb	12.4	5.1	4.3
Average torque	mNm	1287.25	560	250

**Table 2:** Design parameters and their values in the conventional and proposed models.

Parameter	HE-PSSFM3	HE-PSSFM2	HE-PSSFM1
Stator slot number	12	12	12
PM height (mm)	1.67	5.6	5.6
Slot package factor	0.5	0.5	0.5
PM thickness (mm)	10	3	3
PM volume (mm${}^{3}$)	2500	5000	2500
Rotor pole-pair number	10	10	10
Inner radius of outer stator (mm)	31.95	31.95	31.95
Rotor inner pole arc	24	24	24
Inner stator inner radius (mm)	10.4	10.4	10.4
Rotor thickness (mm)	3.5	3.5	3.5
Rated speed (rpm)	400	400	400
Inner stator outer radius (mm)	27.45	27.45	27.45
Air-gap length (mm)	0.5	0.5	0.5
Active axial length (mm)	25	25	25
Inner stator yoke radius	7.5	16.5	16.5
Outer radius stator (mm)	45	45	45

**Table 3:** Machine torque characteristics.

		HE-PSSFM3			HE-PSSFM1			HE-PSSFM2		
Field Winding	Unit	0	Js = −5	Js = 5	0	Js = 5	Js= −5	0	Js = 5	Js= −5
Tavg	Nm	1.29	1.25	1.34	1.0	1.12	0.87	1.08	1.28	1.0012

**Table 4:** Parameters of initial and final design.

Parameter	Unit	Initial Design	Optimize Design
Stator slot number	Not exist	12	12
PM height	mm	1.67	1.67
Slot package factor	Not exist	0.5	0.5
PM thickness	Mm	10	10
PM volume	mm${}^{3}$	2500	2500
Stator yoke radius	mm	43	41
Active axial length	mm	25	25
Rotor inner pole arc	deg.	24	16.5
Rated speed	r/min	400	400
Rotor outer pole arc	deg.	25	24
Rotor radial thickness	mm	3.5	3.5
Inner stator outer radius	mm	27.45	27.45
Inner stator inner radius	mm	10.4	10.4
Rotor pole-pair number	Not exist	10	10
Outer Stator inner radius	mm	31.95	31.95
Air-gap length	mm	0.5	0.5
Stator outer radius	mm	45	45
